# Expression of Selenoproteins Is Maintained in Mice Carrying Mutations in SECp43, the tRNA Selenocysteine 1 Associated Protein (*Trnau1ap*)

**DOI:** 10.1371/journal.pone.0127349

**Published:** 2015-06-04

**Authors:** Yassin Mahdi, Xue-Ming Xu, Bradley A. Carlson, Noelia Fradejas, Paul Günter, Doreen Braun, Eileen Southon, Lino Tessarollo, Dolph L. Hatfield, Ulrich Schweizer

**Affiliations:** 1 Institut für Biochemie und Molekularbiologie, Rheinische Friedrich-Wilhelms-Universität Bonn, Bonn, Germany; 2 Mouse Cancer Genetics Program, Center for Cancer Research, National Cancer Institute, National Institutes of Health, Bethesda, Maryland, United States of America; Montana State University, UNITED STATES

## Abstract

Selenocysteine tRNA 1 associated protein (*Trnau1ap*) has been characterized as a tRNA^[Ser]Sec^-binding protein of 43 kDa, hence initially named SECp43. Previous studies reported its presence in complexes containing tRNA^[Ser]Sec^ implying a role of SECp43 as a co-factor in selenoprotein expression. We generated two conditionally mutant mouse models targeting exons 3+4 and exons 7+8 eliminating parts of the first RNA recognition motif or of the tyrosine-rich domain, respectively. Constitutive inactivation of exons 3+4 of SECp43 apparently did not affect the mice or selenoprotein expression in several organs. Constitutive deletion of exons 7+8 was embryonic lethal. We therefore generated hepatocyte-specific *Secp43* knockout mice and characterized selenoprotein expression in livers of mutant mice. We found no significant changes in the levels of ^75^Se-labelled hepatic proteins, selenoprotein levels as determined by Western blot analysis, enzymatic activity or selenoprotein mRNA abundance. The methylation pattern of tRNA^[Ser]Sec^ remained unchanged. Truncated *Secp43^ Δ7,8^*mRNA increased in *Secp43*-mutant livers suggesting auto-regulation of *Secp43* mRNA abundance. We found no signs of liver damage in *Secp43*3-mutant mice, but neuron-specific deletion of exons 7+8 impaired motor performance, while not affecting cerebral selenoprotein expression or cerebellar development. These findings suggest that the targeted domains in the SECp43 protein are not essential for selenoprotein biosynthesis in hepatocytes and neurons. Whether the remaining second RNA recognition motif plays a role in selenoprotein biosynthesis and which other cellular process depends on SECp43 remains to be determined.

## Introduction

Selenoproteins are proteins containing selenocysteine (Sec), the 21^st^ proteinogenic amino acid. Incorporation of Sec into protein requires the recoding of UGA codons in both eukaryotes and prokaryotes, although the details differ among eubacteria, archeae, and eukaryotes [1, 2]. Selenoproteins occur in all three of the life kingdoms, Bacteria, Archaea and Eukaryota. Another expansion to the genetic code is pyrrolysine, the 22^nd^ proteinogenic amino acid, which has been found in only several archaea and eubacteria, including one human pathogen, and is incorporated into protein by recoding of UAG [3]. How exactly the expansion of the genetic code occurred by use of termination codons is an interesting phenomenon, because in both instances UGA and UAG also retained their functions as termination codons [4]. For example, in the case of Sec insertion into protein, the selenoprotein biosynthetic machinery encodes Sec insertion sequence (SECIS) elements, which are cis-acting RNA motifs, that occur immediately following the Sec UGA in eubacteria, or are positioned in the 3’-untranslated region in eukaryotes and archeae of the mRNA, to distinguish elongation from termination function [5]. SECIS element binding proteins interact directly or indirectly with the translation machinery dictating the insertion of Sec into the growing polypeptide chain, e.g., SECIS binding protein 2 (Secisbp2) in mammals. Sec-tRNA^[Ser]Sec^ interacts with a specific translation elongation factor, designated SelB in bacteria and EF-Sec in archaea and eukaryotes, but not with the EF-Tu or EF-1α. The biosynthetic pathway of Sec initiates with the attachment of serine to tRNA^[Ser]Sec^ by seryl-tRNA synthetase (Serrs) to form Ser-tRNA^[Ser]Sec^, which is further phosphorylated by the phosphoserine tRNA kinase (Pstk). Biosynthesis of Sec-tRNA^[Ser]Sec^ is completed by selenophosphate synthetase 2 (Sephs2), a selenoprotein in many higher animals, that synthesizes the active donor of selenium, selenophosphate, and Sec synthase. This enzyme is designated SelA in bacteria [6], but the eukaryotic protein received several names, soluble liver antigen (SLA) [7], SecS [8], and Sepsecs [9], the latter being adopted as the systematic gene name. A hierarchy of Sec insertion has been described among selenoproteins which may correlate with the affinity of Secisbp2 to SECIS elements [10, 11] and/or with the modification status of tRNA^[Ser]Sec^ (reviewed in [12]). Transfer RNA^[Ser]Sec^ exists in higher animals in two isoforms which differ by the occurrence of a 2’-*O*-hydroxymethyl group (Um34) on the ribosyl moiety of the highly modified base 34, methylcarbonylmethyl-uridine (mcmU_34_). In the presence of amply available selenium (Se), mcmU_34_ is further modified to Um34 [1]. Another modification which affects translation efficiency and possibly tRNA-protein interactions is isopentenylation of A_37_ [13].

SECp43 was initially cloned in a degenerate PCR screen for RNA-binding proteins [14]. The protein harbors two RNA recognition motifs (RRM) and a C-terminal Tyr-rich domain of unknown function. Affinity purification of the native protein with an antiserum generated against recombinant protein co-purified a 90 nt RNA which was subsequently identified by direct RNA sequencing as tRNA^[Ser]Sec^ [14]. It was thus proposed that SECp43 has a role in selenoprotein biosynthesis.

SECp43 further interacted with a 48 kDa protein [7], which was subsequently identified as Sepsecs. Knockdown of SECp43 and Sepsecs reduced selenoprotein expression in cultured NIH 3T3 cells [15]. Protein levels of glutathione peroxidase 1 (Gpx1) were reduced as measured by Western blot analysis and the fraction of methylcarbonylmethyl-uridine-Um34 (mcmU_34_m) tRNA^[Ser]Sec^ compared to mcmU_34_ tRNA^[Ser]Sec^ was reduced in NIH 3T3 fibroblasts [15]. Finally, co-immunoprecipitation indicated binding of SECp43 to Sepsecs [15]. The interaction of SECp43 with purified, fully modified [^75^Se]-Sec-tRNA^[Ser]Sec^ bound to recombinant EF-Sec was examined [16]. Again, SECp43 did not interact with Sec tRNA^[Ser]Sec^, but reduced the electrophoretic mobility of a complex containing [^75^Se]-Sec-tRNA^[Ser]Sec^ and EF-Sec, albeit independent of GTP [16]. Based on the clear effects of SECp43 knockdown on selenoprotein expression in cultured cells, we decided to test the role of SECp43 in selenoprotein expression *in vivo* by gene targeting. Due to the organization of the genomic locus, two mutants were generated which led in both cases to *in-frame* deletions in the protein. Whereas constitutive deletion of exons 3 and 4 neither affected selenoprotein expression nor embryonic development, constitutive deletion of exons 7 and 8 was embryonic lethal. Because inactivation of the tRNA^[Ser]Sec^ gene (*Trsp*) [17] or *Secisbp2* [18] in mice likewise is embryonic lethal, we conditionally inactivated SECp43 in hepatocytes and neurons and extensively analyzed hepatic and cerebral selenoprotein expression.

## Materials and Methods

### Gene targeting and mice

The targeting vector for *SECp43* ^*Δ3*,*4*^ was made by inserting the genomic fragment spanning exons 3 and 4 (1.2 kb) between two *loxP* sites using *Afl* II and *Mfe* I restriction sites, followed by a *loxP*- and *FRT*-flanked neomycin phosphotransferase expression cassette. A 3.1 kb of 5’-homologous sequences upstream and a 6.5 kb of 3’-homologous sequence downstream of the exons 3 and 4 were inserted before and after the *loxP* sites in the vector to guide the homologous integration (S1 Fig). Constitutive deletion of exons 3 and 4 (*Secp43* ^*Δ3*,*4*^) by crossing with *EIIA-Cre* mice did not affect the phenotype of mice in any recognizable way and did not reduce ^75^Se incorporation in liver, kidney, testes, lung, heart, brain and plasma (S2 Fig). This mouse line was not further analyzed.

**Fig 1:**
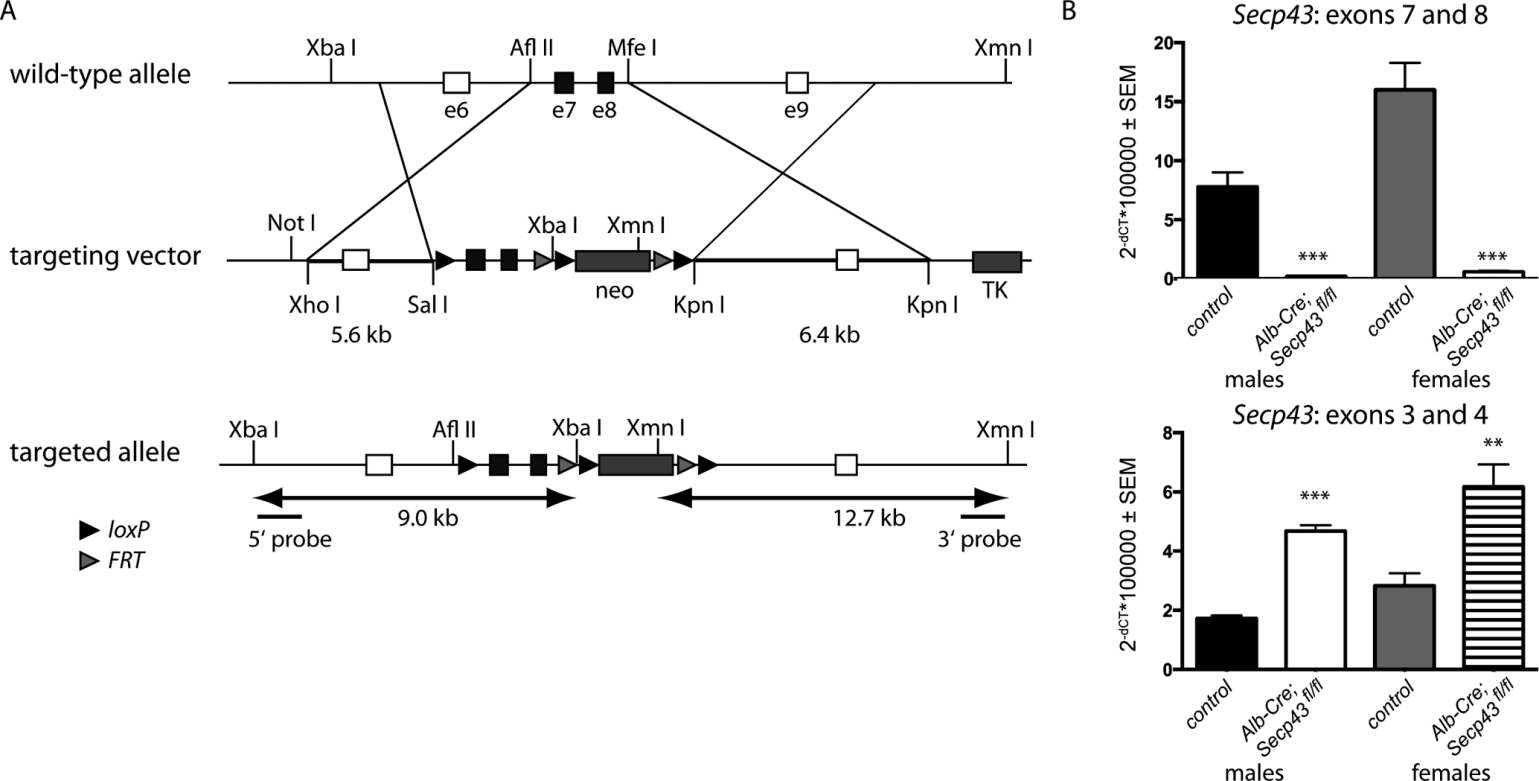
Liver-specific removal of *Secp43* in mice. (A) Schematic representation of gene targeting of *Secp43* in embryonic stem cells. The neomycin cassette (neo) was removed leaving exons (e) 7 and 8 flanked by *loxP* elements. Correct targeting was verified by Southern Blot using the indicated restriction enzymes and probes. (B) Hepatic *Secp43* mRNA levels assessed by qPCR. Deletion of exons 7 and 8 was virtually complete in livers of *Alb-Cre; Secp43* ^*fl/fl*^ mice (***p<0.001), while the abundance of the truncated mRNA increased as shown with primers directed against exons 3 and 4 (***p<0.001; **p<0.01). Student’s *t* test. N = 4–7 per group.

The targeting vector for *Secp43* ^*Δ*7,8^ was prepared using the same backbone vector as the *Secp43* ^*Δ3*,*4*^ knockout and contained a *lox*P flanked genomic fragment spanning exons 7 and 8 and adjacent intronic sequence followed by a *loxP*- and *FRT*-flanked neomycin phosphotransferase expression cassette. A 5.5 kb of 5’-homologous sequences upstream and a 6.4 kb of 3’-homologous sequence downstream of the exons 7 and 8 were inserted before and after the *loxP* sites in the vector (Fig 1A) to guide the homologous integration. The linearized targeting vectors were electroporated into embryonic stem cells. Correct gene targeting was ascertained by Southern blotting with external probes as indicated in Fig 1A. The modified allele was transmitted through the germline and the selection cassette deleted by *FLPe*-mediated recombination. The resulting *loxP*-flanked conditional allele is designated *Secp43* ^*fl*^, the recombined deleted allele is designated *Secp43* ^*Δ7*,*8*^. The mice were backcrossed into a C57Bl/6 genetic background over several generations. Genotyping was done by PCR using the following primers, flSecp43fwd, 5’-catgtgggtcagggatcttc-3’, and flSecp43rev, 5’-caaatgctaatcaaaagatgtca-3’, which spanned the *loxP*-element in intron 8. The wild-type and floxed alleles yield products of 310 bp and 410 bp, respectively. Constitutive knockouts were produced using *EIIA-Cre* [19]. Hepatocyte-specific and neuron-specific knockouts were produced with *Alb-Cre* (albumin promoter-Cre) and *T*α*1-Cre* (tubulin α1promoter-Cre) mice as described [18, 20]. Mice were fed breeding diet (Ssniff, Soest, Germany) containing on average 0.2–0.3 ppm Se. ^75^Se (specific activity ~1000 Ci/mmol) was obtained from the University of Missouri Research Reactor Center (Columbia, MO, USA) and metabolic labeling was performed as described [21]. Mice were handled in accordance with the National Institutes of Health Institutional Guidelines (NCI, NIH, Bethesda, MD, USA), and all mouse experiments were approved by the Animal Ethics Committee at the National Institutes of Health. Mutant mouse lines and other renewable research material are freely available to non-profit researchers upon request to the authors. The ARRIVE statement is found in S1 Text.

### Selenium determination in serum

Se was determined by total reflection X-ray fluorescence (TXRF) using a Picofox S2 instrument (Bruker nano, Berlin, Germany) [22]. Gallium was used as an internal standard for quantification. Reference samples for serum (Sero, Billingstad, Norway) were included in all analyses and results were always within the reference range. All samples were measured in duplicate.

### Liver transaminase determination

Alanine-aminotransferase (ALAT, GPT) and aspartate-aminotransferase (ASAT, GOT) measurements in serum were performed according to standard coupled tests involving 2-ketoglutarate and NADH plus alanine and lactate dehydrogenase (for ALAT) or aspartate and malate dehydrogenase (for ASAT). The decrease in A_340_ was followed over three minutes and the activity was calculated as 1 unit = 1μmol/min from the slope of the linear part of the curve [18].

### Selenoenzyme measurements

Gpx assays were carried out with *tert*-butyl hydroperoxide and thioredoxin reductase (Txnrd) activities were assessed with the DTNB assay in cytosolic fractions of tissue homogenates as described [23]. Protein concentrations were determined by the method of Bradford using IgG as a standard. Type I-deiodinase (Dio1) activity was determined as described with ^125^I-rT_3_ as substrate [24]. 15 μg of membrane fraction protein was used with 1 μM unlabeled rT_3_. The reaction time was 60 min and the reaction temperature 37°C. The assay was done in triplicate and repeated one additional time with similar results.

### Antibodies and Western blot analysis

The antiserum directed towards Sepp (1:400) was generated in rabbits by immunization with a synthetic C-terminal peptide (ImmunoGlobe, Himmelstadt, Germany) and its specificity verified using wild-type and *Sepp*-knockout mice [22, 25]. For serum Sepp quantification, 0.2 μl serum was applied per lane. 25–100 μg protein from the cytosolic fraction were separated on SDS/12% polyacrylamide gels. Antibodies against Sepk and Seps were from Sigma (ATLAS Prestige antibodies, rabbit polyclonal) and used at 1:500, 1:1000 dilution, respectively. Rabbit polyclonal antibodies against Gpx1 and Gpx4 were both from Abcam (Cambridge, UK) and were used at 1:2000 and 1:1000, respectively. The polyclonal rabbit Sepsecs antiserum (Sigma, München, Germany) was used at 1:1000 dilution. Chicken polyclonal antibodies directed towards SECp43 were kindly provided by Dr. Paula Grabowski (University of Pittsburgh) and used at a 1:1000 dilution. After electrotransfer, nitrocellulose membranes were stained with Ponceau Red, photographed and blocked with 5% BSA for 1 hour at 25°C. Rabbit polyclonal *β*-actin antiserum (Sigma, Munich, Germany) was used as a loading control at 1:2000 dilution.

### qPCR analysis

Total RNA was isolated from powdered mouse liver according to the TRIzol protocol (Invitrogen, Darmstadt, Germany). Samples were treated with RQ1 RNase-Free DNase (Promega, Madison, USA). cDNA was synthesized using the iScript cDNA synthesis kit (Biorad, München, Germany) according to the manufacturer's protocol. qPCR was performed using SYBR Green from Abgene (Thermo Scientific, Epsom, UK) on a Mastercyler epgradient S realplex (Eppendorf, Germany). Primers used for qPCR detection of selenoproteins genes were as described previously [18]. Primers used in this study for the first time are: Serrs fwd: GAATATTGTCTCAGGCTCCTTG, Serrs rev: GGGTGGTAGCACACATTGTAG, SECp43 e7,8 fwd: ACCAGAACTACTATGCCCAGTG, SECp43 e7,8 rev: ATTATTCTAGGCCTCTGCCTTC, SECp43 e3,4 fwd: TGCATAAAATTAATGG GAAACC, SECp43 e3,4 rev: AGGGAGTACTCAGGGCTATTGT, Sepsecs fwd: CTACAGGAAGC TGTTGAAGGAG, Sepsecs rev: TTTGTCATGGTGTCCATCTATC, Pstk fwd: CATGTTTGAAGA GGAATGGTGA and Pstk rev: TCCAATGCAGTAAGCAACAAAC. 18S rRNA was used as the reference gene for mRNA quantification.

### Isolation, fractionation and quantitation of tRNA

Total tRNA was isolated from mouse liver of control and *Alb-Cre; SecP43* ^*fl/fl*^ mice, and the amounts of tRNA^Sec^ were quantified following TBE-Urea gel electrophoresis and hybridization with a ^32^P-labelled probe by Northern blot analysis using a PhosphorImager (Molecular Dynamics) [19]. Total tRNA from control and *Alb-Cre; SecP43* ^*fl/fl*^ mice was aminoacylated with [^14^C]serine with [^3^H]serine, respectively, in the presence of rabbit reticulocyte synthetases. The resulting aminoacylated tRNA was fractionated on an RPC-5 column [26], first in the absence and then in the presence of Mg^2+^ as given [19, 21].

### Rotarod assay

Movement co-ordination was assessed as described previously [27].

### Immunohistochemistry

Mouse brains were immersion fixed in 4% paraformaldehyde in 0.1 M phosphate buffer (PB), pH 7.4. Over night fixation of the brains at 4°C was followed by cryoprotection in 30% sucrose in 0.1 M PB for 2 days, and the brains were frozen at -80°C. Sections were cut at 20 μm on a cryostat. Free floating sections were stained with polyclonal rabbit α-calbindin (Swant, Bellinzona, Switzerland) at a dilution of 1:10.000 and developed with horseradish peroxidase-anti rabbit conjugate [20]. Additional methods are found in S2 Text.

### Statistics

For all computations, GraphPad Prism 6 for Mac OS X software was used for the tests indicated in the figure legends. Data are expressed as means ± standard error of the mean (S.E.M.). Statistical significance was determined and indicated as **p < 0.01, or ***p < 0.001.

## Results

### Genomic manipulation of the *Tranu1ap* locus in mice

The gene encoding SECp43, *Trnau1ap*, consists of 9 exons and is located on chromosome 1. The many small exons and their arrangement made it difficult to choose exons for gene targeting. We selected two conditional constructs, one of which deleted exons 3 and 4 eliminating portions of the first RRM (*Secp43* ^*Δ*3,4^), and the other that deleted exons 7 and 8 eliminating a portion of the Tyr-rich domain (*Secp43* ^*Δ*7,8^) (Fig 1A). Both constructs had the drawback that the deletions were in-frame.

Constitutive *Secp43* ^*Δ*3,4/ *Δ*3,4^ mice showed no apparent phenotype and accordingly no change in selenoprotein expression. Construction and characterization of these mice are described in S1, S2 and S3 Figs. A Western blot using antiserum against SECp43 revealed a smaller band of SECp43 that was highly expressed in liver (S3 Fig) and likely corresponded to the product of an mRNA lacking exons 2 and 3.

To target the Tyr-rich domain, we introduced *loxP* elements flanking exon 7 and 8 leading to a *Cre*-responsive conditional allele, designated *Secp43* ^*fl*^ (Fig 1A). Germline deletion of both exons resulted in embryonic lethality as observed among 192 offspring from heterozygous matings (*Secp43* ^*+/ Δ7*,*8*^ x *Secp43* ^*+/ Δ7*,*8*^), wherein 69 *Secp43* ^*+/+*^ and 123 *Secp43* ^*+/ Δ7*,*8*^ mice were found, but none with a *Secp43* ^*Δ7*,*8 / Δ7*,*8*^ genotype. The numbers are compatible with Mendelian inheritance and embryonic lethality. Since we expected an essential function of SECp43 for selenoprotein expression and since *Trsp*- and *Secisbp2*-deficient mice also die early during embryonic development, we did not determine the exact phenotype of the homozygous knockout embryos, but proceeded to conditional inactivation of SECp43 in liver.

Hepatocyte-specific inactivation of *Secp43* was achieved by *Cre*-mediated recombination using *Alb-Cre* transgenic mice. Western blot analysis of SecP43 revealed the loss of SECp43 in liver of *Alb-Cre; Secp43* ^*fl/fl*^ mice, but not in kidney or testes, which were used as control tissues (S4 Fig). Quantitative deletion of exons 7+8 in *Secp43* was demonstrated by qPCR using primers located in exons 7 and 8. Virtually no transcript containing exons 7 and 8 was detected in *Alb-Cre; Secp43* ^*fl/fl*^ liver (Fig 1B). In contrast, when using primers located in exons 3 and 4, *Secp43* ^*Δ7*,*8*^, mRNA levels were apparently increased in mutant liver (see below).

We then tested whether liver damage occurred in *Secp43*-mutant mice. Liver transaminase activities were determined in plasma from adult mice, but no significant increase in transaminase activity was noted in *Secp43-*mutant mice (Table 1). This result suggested that the mutation did not impair the integrity of the liver.

**Table 1:** Plasma activities of liver transaminases.

		controls	*Alb-Cre; Secp43* ^*fl/fl*^
ASAT/GOT	♂	11.1 ± 4.9 U/L	17.3 ± 7.1 U/L
	♀	18.6 ± 14 U/L	26.1 ± 10.8
ALAT/GPT	♂	9.4 ± 3.3 U/L	13.7 ± 5.7 U/L
	♀	7.8 ± 3.3 U/L	13.3 ± 6.7 U/L

Mean values ± SEM are given. Numbers of animals: male controls (5), male mutants (4), female controls (7), and female mutants (6). Although the mean plasma transaminase values appeared to increase, the differences were not significant (Student’s *t*-test) and only values above 50 U/L would be regarded pathological.

### ^75^Se-metabolic labeling in Secp43 ^Δ7,8^ mutant mice

**Fig 2:**
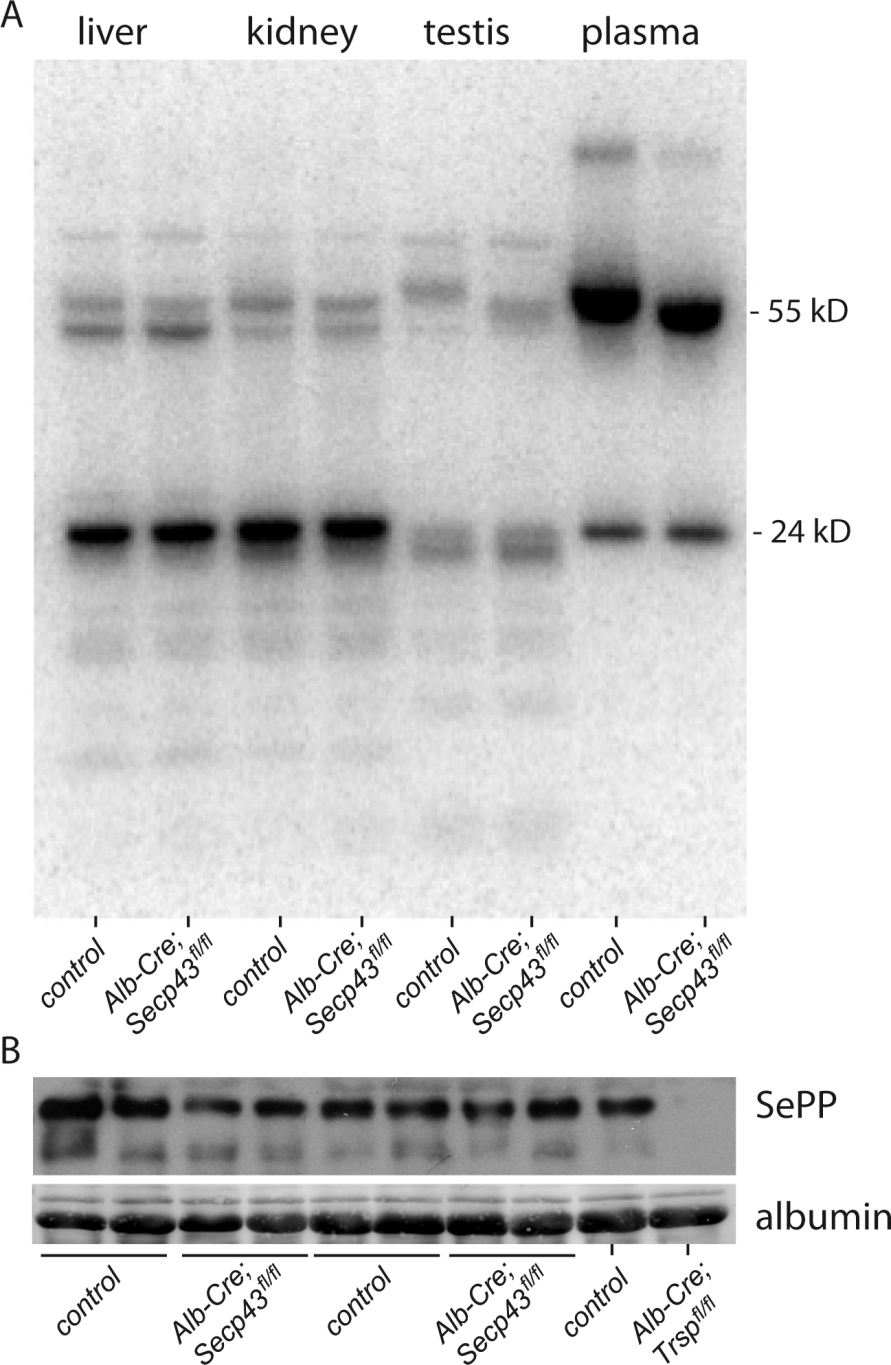
Selenoprotein expression in organs and plasma from *Alb-Cre; Secp43* ^*fl/fl*^ mice. (A) Mice were labeled with ^*75*^Se and proteins extracted from organs were separated on a polyacrylamide gel. (B) Selenoprotein P in plasma visualized by Western blotting. Plasma from *Alb-Cre; Trsp* ^*fl/fl*^ mouse was used as negative control. Equal protein loading was ascertained by Ponceau S staining of the blotting membrane.

Incorporation of Se into selenoproteins can be easily visualized by metabolic labeling of mice with ^75^Se-selenite. Fig 2A shows an autoradiogram of liver, kidney, testis, and plasma proteins resolved by SDS-PAGE. Samples from a wild-type litter mate and an *Alb-Cre; Secp43* ^*fl/fl*^ mouse were compared. ^75^Se-labeling of selenoproteins in liver of *Secp43*-mutant and control mice was virtually identical. This finding indicated that at least the most abundant selenoproteins (Txnrd1, Gpx1, Gpx4, Dio1, and other, not unequivocally assigned, selenoproteins) incorporated normal amounts of selenium in *Secp43* knockout hepatocytes. Plasma selenoproteins, selenoprotein P (Sepp) and Gpx3, incorporated similar amounts of ^75^Se in both control and *Secp43* mutant mice. An apparent size difference in Sepp was noted in an initial experiment between control and *Secp43* mutant mice, but the difference was not apparent by Western blotting from additional mice that were further backcrossed into the C57Bl/6 genetic background (Fig 2B). Accordingly, plasma selenium levels were not different between *Alb-Cre; Secp43* ^*fl/fl*^ mice and their littermate controls (Table 2).

**Table 2:** Plasma selenium content.

	controls	*Alb-Cre; Secp43* ^*fl/fl*^
males	435 ± 20 μg/L	464 ± 10 μg/L
females	421 ± 16 μg/L	426 ± 13 μg/L

Mean values ± SEM are given. Numbers of animals: male controls (5), male mutants (5), female controls (7), and female mutants (6). No significant differences were found (Student’s *t*-test).

### Hepatic selenoprotein expression in Secp43 ^Δ7,8^ mutant mice

**Fig 3:**
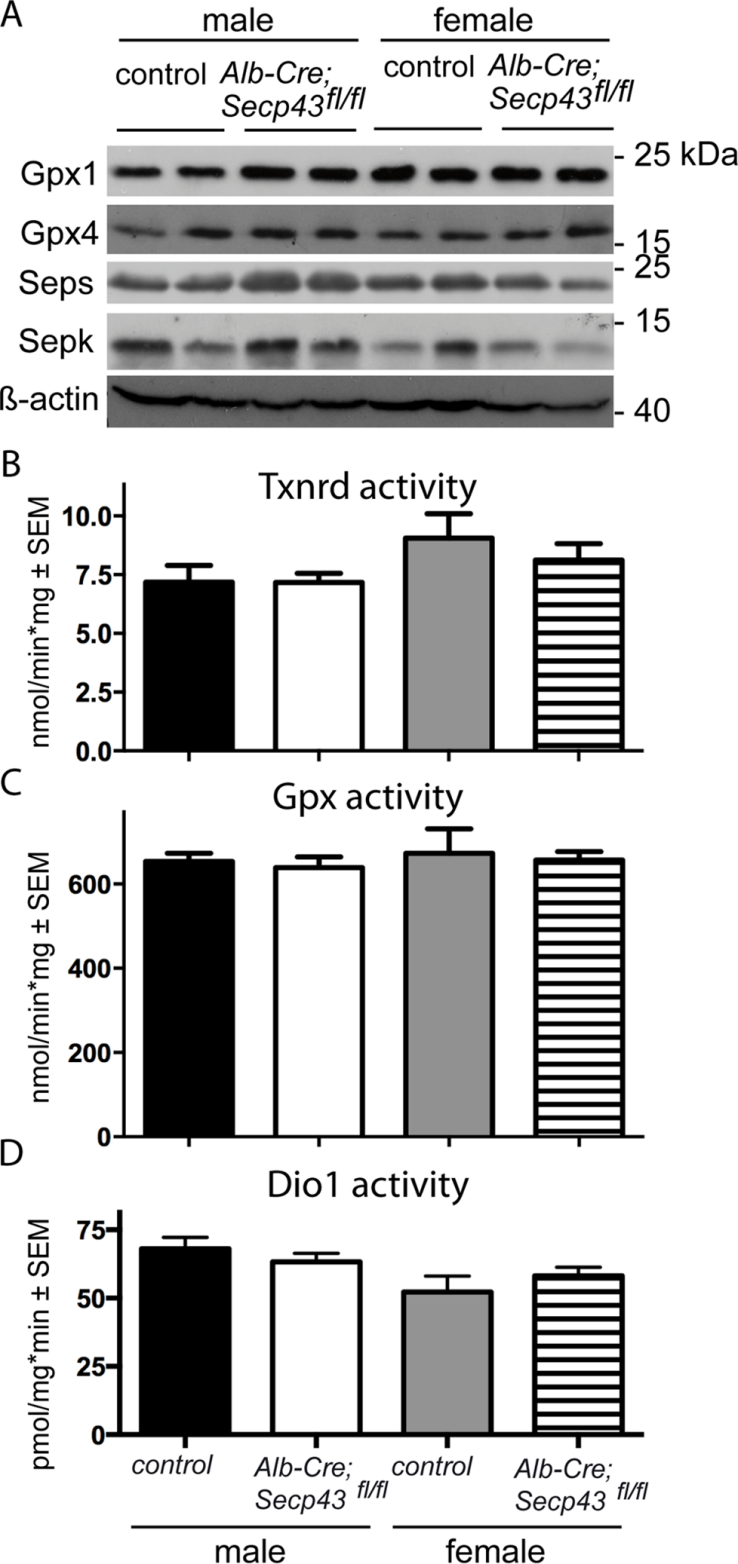
Selenoprotein expression and selenoenzyme activities in liver of *Alb-Cre; Secp43* ^*fl/fl*^ and control mice. (A) Selenoprotein levels were assessed by Western blotting and show no differences in *Secp43*-mutant mice. (B) Cytosolic Txnrd activity. Number of animals: males (n = 4–5), females (n = 6–7). (C) Cytosolic Gpx activity. Number of animals: males (n = 4), females (n = 6–7). (D) Dio1 activity. Number of males (n = 6–7), females (n = 7–12).

Sepp is the main plasma selenium carrier protein predominantly made by hepatocytes [25]. Biosynthesis of nascent Sepp polypeptide and increasingly glycosylated Sepp were not changed in livers from *Secp43*-deficient mutant as assessed by Western blotting (not shown). Expression of selenoproteins which can be assigned in ^75^Se autoradiographs (Gpx1 and Gpx4) were also assessed by Western blot analysis. Again, the amounts of Gpx1 and Gpx4 were not reduced in *Alb-Cre; Secp43* ^*fl/fl*^ liver. Likewise, levels of selenoproteins K and S (Sepk and Seps, respectively) were not changed (Fig 3A). Taken together, these results indicate that selenoprotein expression in hepatocytes was not impaired by loss of the essential Tyr-rich domain in *SECp43*.

We then tested enzymatic activities of selenoenzymes which can be readily assessed. Again, no differences were observed in the activities of cytosolic thioredoxin reductase (Txnrd), cytosolic glutathione peroxidase, and deiodinase 1 (Dio1) in livers from *Secp43*-mutant mice (Fig 3B–3D).

### mRNA levels of hepatic selenoproteins and selenoprotein biosynthesis factors

**Fig 4:**
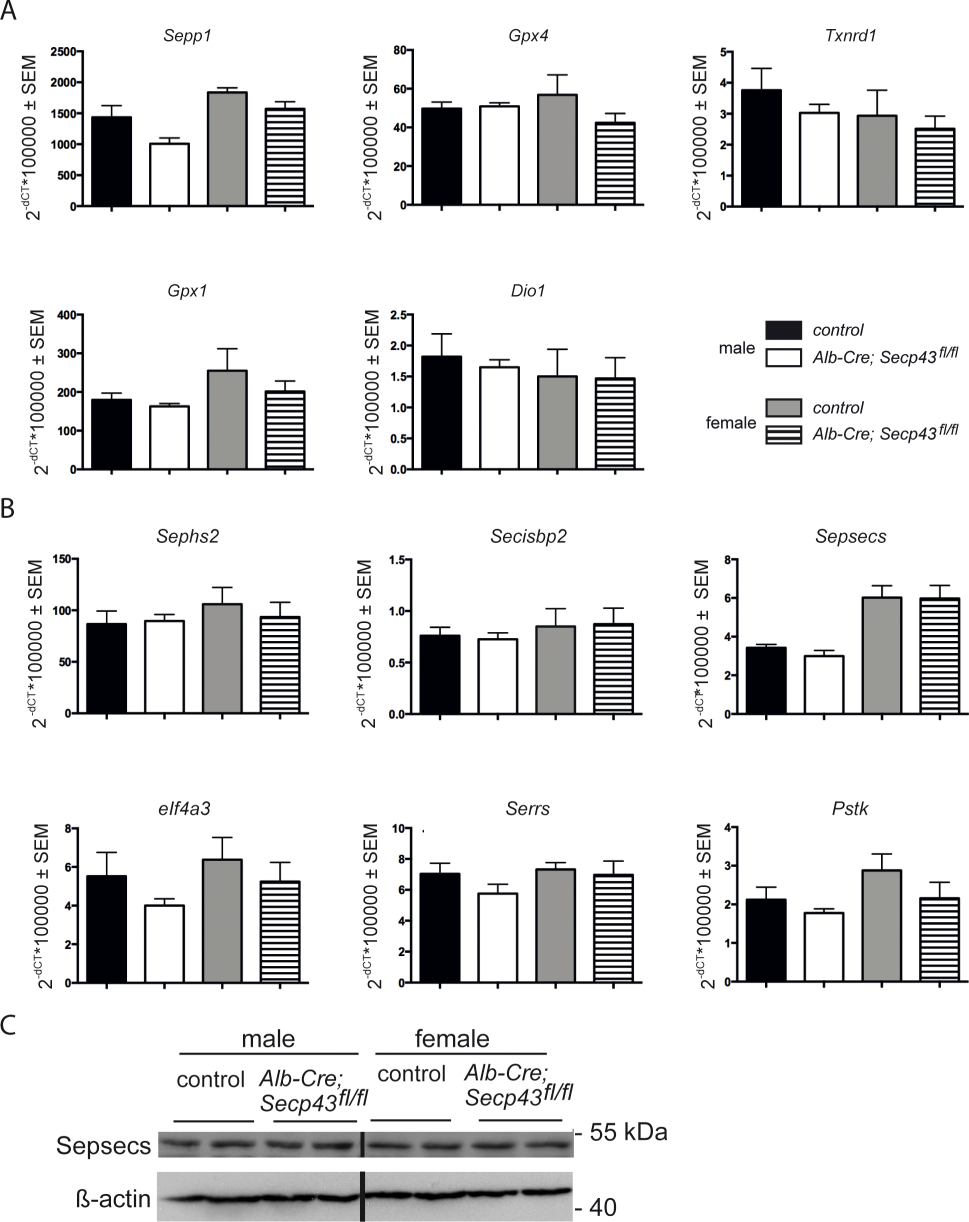
Expression of selenoprotein and selenoprotein factor mRNAs activities and Sepsecs in liver of *Alb-Cre; Secp43* ^*fl/fl*^ and control mice. (A) Selenoprotein mRNA levels were determined by qPCR. *18S* rRNA served as control. No significant differences were observed between genotypes (Student’s *t* test). Number of animals: male controls (n = 5), male mutants (n = 4), female controls (n = 4–6), female mutants (n = 6–7). (B) Selenoprotein biosynthesis factor mRNA levels were determined by qPCR. *18S* rRNA served as control. No significant differences were observed between genotypes (Student’s *t* test). Number of animals: male controls (n = 5), male mutants (n = 4), female controls (n = 4–6), female mutants (n = 6–7). (C) Sepsecs levels were determined by Western blotting. A protein of 48 kDa was detected in liver. No change was observed in the *Secp43* knockout.

We speculated that even if SECp43 may not be an essential component of selenoprotein expression in liver, this protein may still gradually facilitate selenoprotein expression, and loss of SECp43 may be compensated by alterations in selenoprotein mRNA levels. We therefore assessed expression of several liver selenoprotein mRNAs by qPCR from *Alb-Cre; Secp43* ^*fl/fl*^ and litter mate control mice. We did not detect any significant differences in mRNA levels encompassing both male and female mice (Fig 4A).

However, SECp43 has also been reported to interact with Sepsecs [14, 15]. Thus, the deficiency in SECp43 may be compensated by an increased expression of Sepsecs and possibly other proteins involved in selenoprotein biosynthesis. We therefore examined mRNA levels of *Serrs*, *Pstk*, *Sepsecs*, and *Sephs2*, which also is a selenoprotein. Very similar mRNA levels were observed for these enzymes (Fig 4B). Moreover, RNA-binding proteins which are known as positive and negative regulators of selenocysteine insertion, *Secisbp2* and *eIF4a3*, respectively, were not changed (Fig 4B). Because of the reported interaction of SECp43 and Sepsecs, we tested Sepsecs expression by Western blotting. This analysis did not reveal any appreciable change in expression of this protein in *Secp43* mutant liver (Fig 4C).

### Methylation status and abundance of tRNA^[Ser]Sec^ in liver

**Fig 5:**
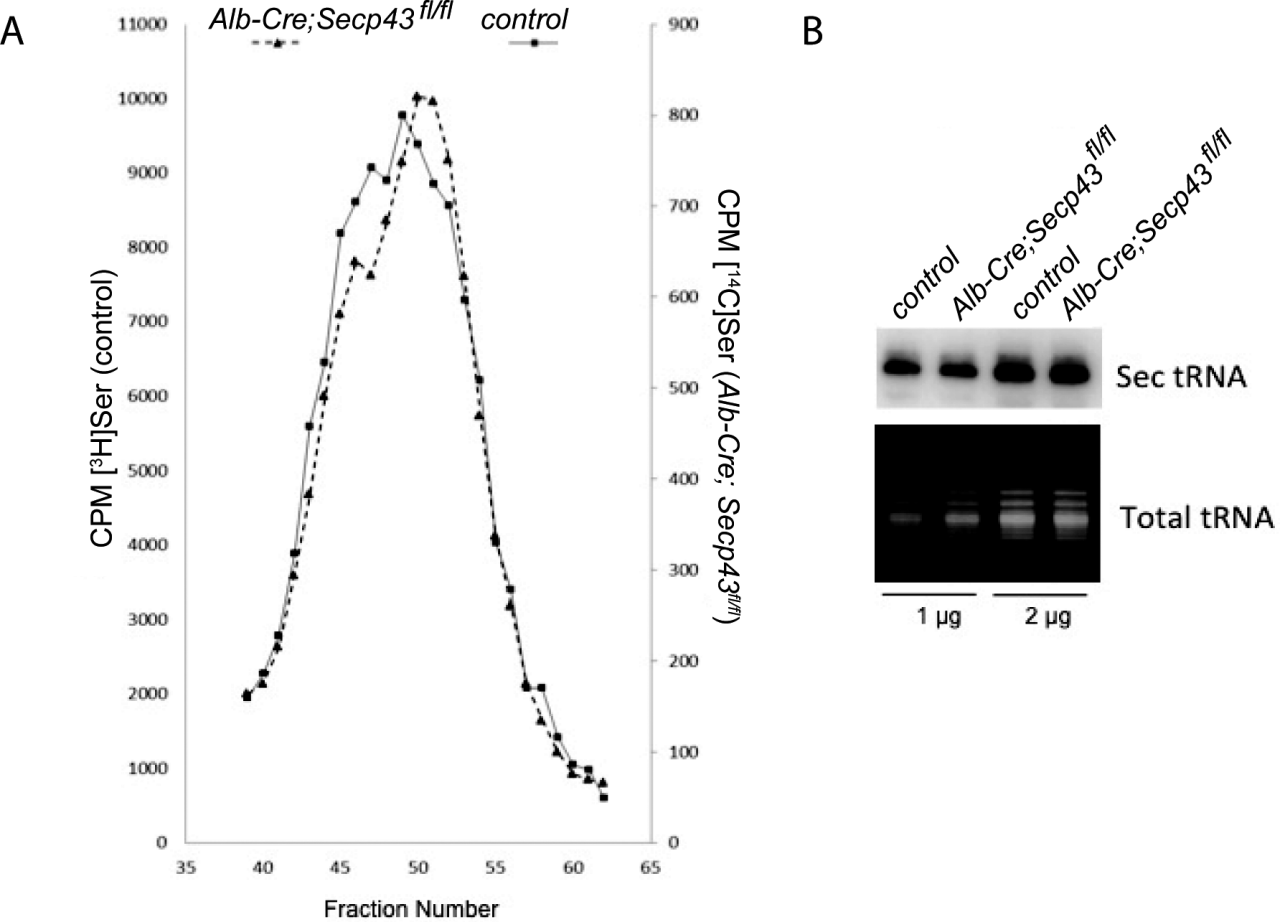
Sec tRNA^*[Ser]Sec*^ expression from liver of *Alb-Cre; Secp43* ^*fl/fl*^ and control mice. (A) Chromatography of seryl-tRNA^*[Ser]Sec*^. Transfer RNA was isolated, aminoacylated with ^*3*^H- or ^*14*^C-serine and co-chromatographed on a RPC-5 column as shown in the figure. (B) Northern blotting of tRNA^*[Ser]Sec*^.

The ratio of mcmU_34_m to mcmU_34_ tRNA^[Ser]Sec^ isoforms responds to Se availability. The methylase involved has not been identified, but siRNA-mediated knockdown of *Secp43* transcript has been shown to lower the fraction of mcmU_34_m in cultured cells [15]. We therefore assessed tRNA^[Ser]Sec^ methylation patterns in livers from *Alb-Cre; Secp43* ^*fl/fl*^ and control mice. The mcmU_34_m isoform is slightly more hydrophobic than the non-Um34 isoform and elutes slightly later from the reverse phase chromatographic column and a reduction of mcmU_34_m would be apparent as a reduction in level at the right-hand flank of the double peak. Virtually no change in the methylation pattern was observed in seryl-tRNA^[Ser]Sec^ from *Secp43*-mutant compared to that from the corresponding control seryl-tRNA^[Ser]Sec^ (Fig 5A). We also quantified the amount of tRNA^[Ser]Sec^ in liver by Northern blotting and found that the levels of total tRNA^[Ser]Sec^ were very similar in the livers of the two mouse lines (Fig 5B).

### Neuron-specific Secp43 mutation

**Fig 6:**
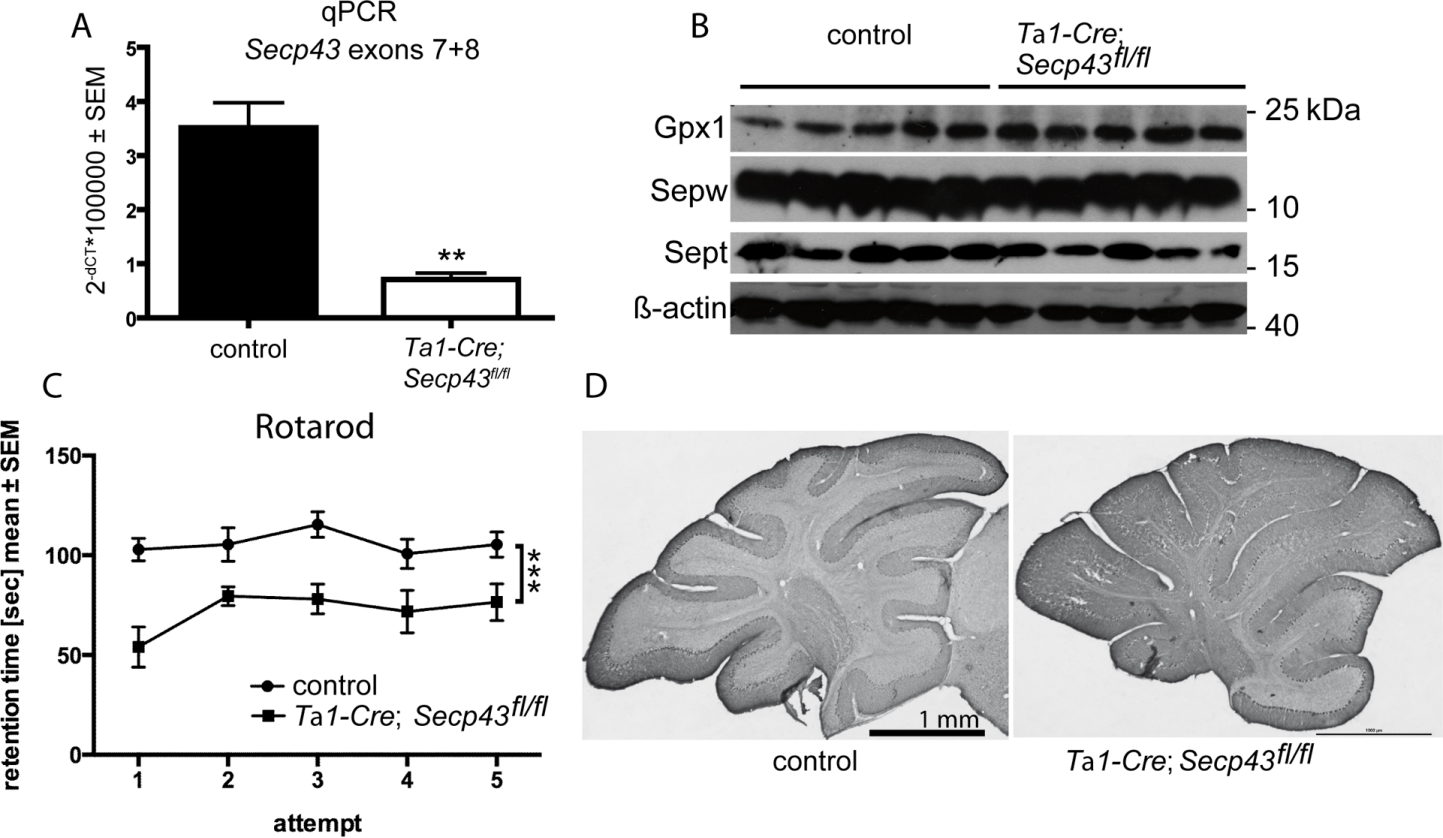
Neuron-specific *Secp43* mutant mice. (A) *Secp43* mRNA levels measured by qPCR. Deletion of exons 7 and 8 was successful in neurons of *T*α*1-Cre; Secp43* ^*fl/fl*^ mice (**p<0.01) Student’s *t* test. Number of animals: male controls (n = 5), male mutants (n = 4). (B) Selenoprotein expression in adult cerebral cortex is not altered as judged by Western blots against stress-related selenoproteins. N = 5 male animals for controls and *T*α*1-Cre; Secp43* ^*fl/fl*^ mice. (C) Rotarod performance is reduced in adult *T*α*1-Cre; Secp43* ^*fl/fl*^ mice. n = 10–16. Significant by genotype, two-way ANOVA. (D) Cerebellar histology is not altered. Normal foliation pattern and calbindin expression in Purkinje cells.

The lack of effect on selenoprotein expression in *Secp43-*mutant hepatocytes prompted us to ask whether this observation is specific for hepatocytes, whereas other cell types may require SECp43. In the brain, SECp43 expression starts during development and is maintained in mature neurons [28]. We therefore conditionally mutated SECp43 in neurons using *T*α*1-Cre* transgenic mice which have been used before in our laboratory to inactivate tRNA^[Ser]Sec^ [20, 29]. *T*α*1-Cre; Secp43* ^*fl/fl*^ mice are born at the expected frequency and show no apparent phenotype. Efficient deletion of exons 7 and 8 in brains of mutant mice was verified by qPCR using primers specific for exons 7 and 8 (Fig 6A). Selenoprotein expression was assessed by Western blotting in brain tissue from *T*α*1-Cre; Secp43* ^*fl/fl*^ and control mice (Fig 6B). We found that the expression of neuronal selenoproteins in *Secp43*-mutant mice compared to the corresponding control mice were very similar.

Movement co-ordination is often impaired in selenoprotein-deficient mice [27, 30, 31]. The performance on the rotarod of *T*α*1-Cre; Secp43* ^*fl/fl*^ mice was significantly reduced compared to normal control animals indicating that the deletion of the Tyr-rich domain in SECp43 has indeed functional consequences (Fig 6C). Cerebellar development is sensitive to disruption of neuronal selenoprotein expression [20, 32]. However, cerebellar foliation and calbindin expression appeared normal in *T*α*1-Cre; Secp43* ^*fl/fl*^ animals (Fig 6D).

Selenoprotein expression is, therefore, independent of the *Secp43* mutation eliminating the Tyr-rich domain in neurons as well as in hepatocytes by the criteria used herein.

## Discussion

### Evidence for a role of SECp43 on selenoprotein expression

SECp43 co-purified with tRNA^[Ser]Sec^ and was reported to interact with a 48 kDa protein which is now believed to represent Sepsecs [14]. It is hard to construct a case in which a low-abundance tRNA of 90 nt, when directly sequenced from the eluate of an affinity column loaded with SECp43, could be misidentified as tRNA^[Ser]Sec^ because of a chance error. The following observation that knockdown of *Secp43* in NIH 3T3 cells changed the methylation status of tRNA^[Ser]Sec^ and reduced the abundance of several selenoproteins was in line with the expectation that SECp43 plays an important role in selenoprotein biosynthesis [15]. Together, these observations were the motivation for conducting the present study in which we wanted to determine the role of SECp43 in selenoprotein biosynthesis *in vivo*, i.e. in transgenic mice.

### Attempts to target Secp43

Our initial attempt to inactivate *Secp43* in mice was by targeting the first RRM by deletion of exons 3 and 4. As described in the supporting information, no effect on selenoprotein biosynthesis was noted in any organ tested and constitutive Secp43 ^*Δ*3,4/ *Δ*3,4^ mice developed normally. Deletion of exons 7 and 8 in mice did not support embryonic development. We initially concluded that embryonic lethality is consistent with defective selenoprotein expression, since *Trsp*- and *Secisbp2*-KO mice also die early during embryonic development [17, 18]. Studying the role of SECp43 in embryonic development was not the aim of the present study. Our goal was to assess the role of SECp43 in selenoprotein biosynthesis, and we therefore generated liver-specific *Secp43* ^*Δ7*,*8*^-mutant mice.

### Effect of Secp43 gene targeting on hepatic selenoprotein biosynthesis

*Alb-Cre; Secp43* ^*Δ7*,*8*^-mutant mice are a good model to study selenoprotein biosynthesis, because hepatocytes express many selenoproteins, but can tolerate the complete abrogation of selenoprotein expression [24, 25]. Lack of Txnrd1 expression induces Nrf2-dependent gene activation and compensatory expression of, e.g., glutathione-S-transferases [18, 33, 34]. Inactivation of Nrf2 combined with inactivation of tRNA^[Ser]Sec^ leads to liver failure [35]. It is also known that inactivation of Txnrd1 causes liver degeneration when glutathione biosynthesis is impaired [36]. No liver damage was observed in *Alb-Cre; Secp43* ^*fl/fl*^ mice as demonstrated by normal liver transaminases in plasma.

Extensive analysis of selenoprotein expression in liver and plasma did not yield any indication that selenoprotein translation is impaired in *Secp43*-mutant liver. Metabolic labeling of selenoproteins with ^75^Se, Western blot of selenoproteins, activity measurements of selenoenzymes, plasma Se content as a marker of liver Sepp production all remained normal in *Alb-Cre; Secp43* ^*fl/fl*^ mice. Selenoprotein mRNAs are sometimes sensitive markers of reduced selenium bioavailability, but also their levels remained normal in *Secp43*-mutant livers. Moreover, there was no compensatory up-regulation of *Sephs2* as e.g., in *Sepp*-deficient mice [37]. EIF4a3, a negative regulator of selenoprotein expression is regulated by Se availability in cells [38], but its mRNA level in liver was not changed in *Secp43*-mutant mice. Because no negative effect on selenoprotein expression was observed, we did not attempt to study expression of Nrf2 targets. Finally, neither tRNA^[Ser]Sec^ abundance nor its methylation status was changed in *Secp43*-mutant liver. In summary, we found no phenotype with respect to selenoprotein expression despite a very extensive analysis.

### Why is there no effect of Secp43 mutations on selenoprotein expression?

One possibility that we did not observe an effect of *Secp43* deficiency on selenoprotein biosynthesis is that the targeted mutations we introduced did not disrupt the function of SECp43. Targeting the *Secp43/Trnau1ap* locus is indeed difficult. The Genbank database contains a transcript lacking exons 2 and 3 (NCBI XM_006539180.1) suggesting that exon skipping occurs *in vivo*. S3 Fig shows that, at least in liver, a smaller form of SECp43 is detected which is indistinguishable from SECp43 ^*Δ*3,4^. Since deletions of exons 2+3, 3+4, and 7+8 maintain the reading frame, it is possible that the truncated proteins retain some of their biological functions. While exons 2–4 encode the first RRM domain, exons 7+8 encode part of the Tyr-rich domain. Our *Secp43* ^*Δ*7,8^ resulting from the *Secp43* ^*fl/fl*^ alleles removes 66 amino acids from a protein of 287 amino acids. This manipulation, in turn, leaves both RRM domains intact. Given the fact that the truncated *Secp43* ^*Δ*7,8^ mRNA is up-regulated rather than degraded, one may argue that our gene targeting strategy did not completely inactivate SECp43, but at least interferes with autoregulation of *Secp43*mRNA levels. The Tyr-rich region is completely conserved among six mammals (human, chimp, dog, cattle, mouse and rat) (S5 Fig). Of note, constitutive *Secp43* ^*Δ7*,*8*^ mice are embryonic lethal clearly indicating that the Tyr-rich domain is important for SECp43 function. This notion is further supported by our observation that movement co-ordination is impaired in neuron-specific *Secp43*-mutant mice and by the finding of developmental expression of SECp43 in the brain [28].

A second possibility is that SECp43 may not be required for selenoprotein biosynthesis in hepatocytes, but in other cell types. This possibility is not supported by our findings in neuron-specific *Secp43*-mutant mice. *T*α*1-Cre; Secp43* ^*fl/fl*^ mice have normal selenoprotein expression in brain. Since cerebellar development [20], cortical interneuron development [29], and striatal interneuron development [31] depend on selenoprotein biosynthesis, and even milder disruption of cerebral selenoprotein biosynthesis [27, 31, 39] causes clear neurological defects, we are confident that we have not missed a mere quantitative reduction of selenoproteins in neural tissues. The animals in our study were fed Se-replete diets. While it may be argued that a potential phenotype could be unmasked by dietary Se restriction, it is nevertheless clear that SECp43 is not essential for selenoprotein biosynthesis in hepatocytes and neurons.

A third possibility is that SECp43 has nothing to do with selenoprotein expression, but has an unrelated role in cells. In a systematic study on the conservation of the selenoprotein biosynthesis pathway in insects, Chapple & Guigo showed that several insect species which have abandoned selenoprotein expression during evolution have lost selenoprotein pathway genes, e.g., selenocysteine synthase or selenophosphate synthase 2 [40]. However, all species, whether they do or do not contain selenoproteins and selenoprotein biosynthesis factors, have retained *Secp43* in their genomes implying that *Secp43* may serve a conserved function independent of selenoprotein expression [40]. Another indication why *Secp43* may not necessarily be associated with selenoprotein expression is the finding that parasitic plant nematodes of the *Tylenchina* clade have lost all selenocysteine incorporation factors, but retained functional *Secp43* genes [41]. Finally, in a reconstituted system for *in vitro* selenoprotein translation, Gupta et al. noted that their wheat germ-based system does not contain SECp43, but was functional as long as Sec-tRNA^[Ser]Sec^, Secisbp2, EF-Sec, and mammalian ribosomes were added [42].

## Supporting Information

S1 Fig Targeting the removal of the SECp43/*Trnaulap* in mice. Schematic representation of targeting exons 3+4 of SECp43/*Trnau1ap*. Details are given in the Experimental Procedures of the main text.
(TIFF)

S2 Fig Selenoprotein expression in liver, kidney, testes, lung, heart, brain and plasma tissues from *Secp43* ^*Δ* 3,4/ *Δ* 3,4^ and control mice. Mice were labeled with ^75^Se, tissues excised, proteins extracted and run on a polyacrylamide gel, the gel dried and exposed using a PhosphorImager (Molecular Dynamics). ^75^Se-labeling of mice and other details are given in the Experimental Procedures of the main text.
(TIFF)

S3 Fig SECp43 western blot analysis in liver, kidney and testes of control, *Secp43* ^*Δ* 3,4/ *Δ* 3,4^ and *Secp43* ^+/ *Δ* 3,4^ mice. Western blot analysis was performed as described in Experimental Procedures of the main text.
(TIFF)

S4 Fig SECp43 western blot analysis in liver, kidney and testes of control and *Alb-Cre; Secp43* ^*fl/fl*^ mice. Western blot analysis was performed as described in Experimental Procedures of the main text.
(TIFF)

S5 Fig Alignment of amino acid sequences within the Tyr-rich domain of SECp43. (TIFF)

S1 Text ARRIVE statement. (PDF)

S2 Text Additional methods. (TIFF)
