# Supplementary figures and images for: Expression of Selenoproteins Is Maintained in Mice Carrying Mutations in SECp43, the tRNA Selenocysteine 1 Associated Protein (Trnau1ap)
Source: PLoS One. 2015 Jun 4;10(6):e0127349. doi: 10.1371/journal.pone.0127349 (PMC4456167; doi:10.1371/journal.pone.0127349)

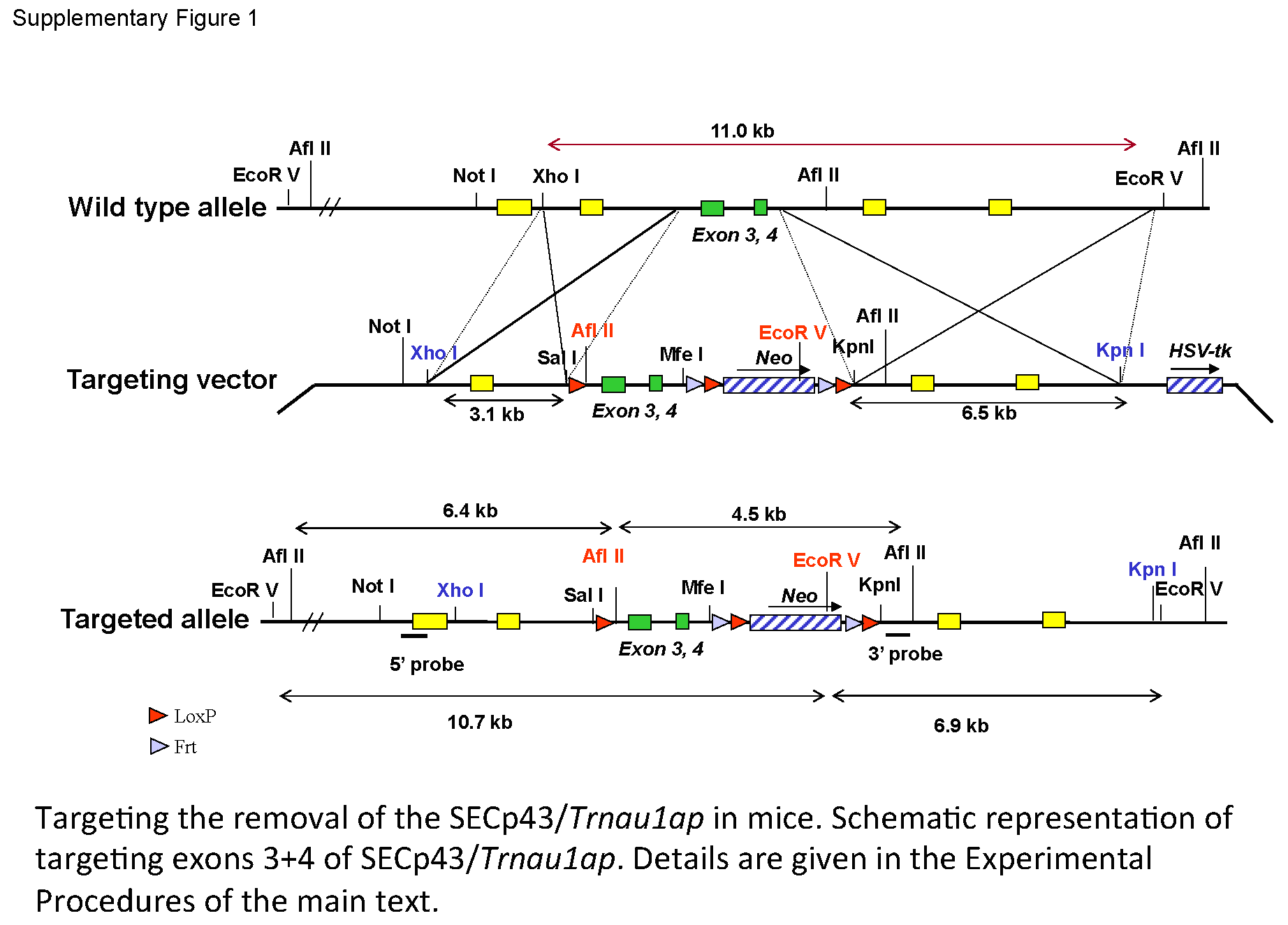

Supplement: S1 Fig — Schematic representation of targeting exons 3+4 of SECp43/Trnau1ap. Details are given in the Experimental Procedures of the main text. (TIFF) [file pone.0127349.s001.tiff]

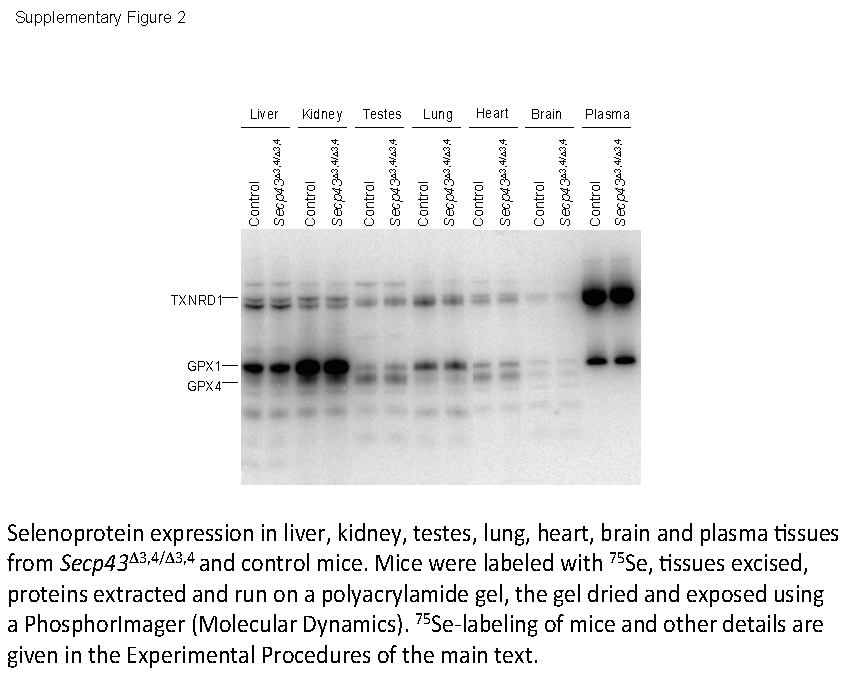

Supplement: S2 Fig — Mice were labeled with 75Se, tissues excised, proteins extracted and run on a polyacrylamide gel, the gel dried and exposed using a PhosphorImager (Molecular Dynamics). 75Se-labeling of mice and other details are given in the Experimental Procedures of the main text. (TIFF) [file pone.0127349.s002.tiff]

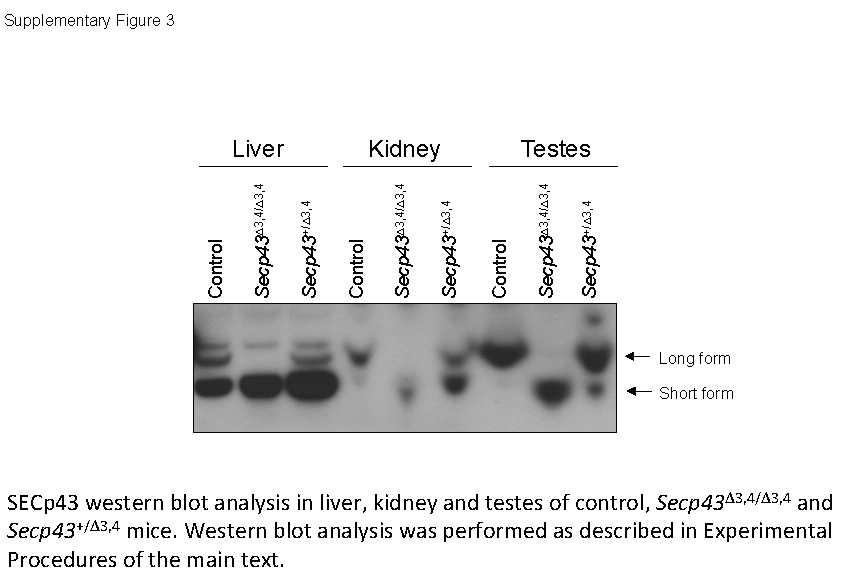

Supplement: S3 Fig — Western blot analysis was performed as described in Experimental Procedures of the main text. (TIFF) [file pone.0127349.s003.tiff]

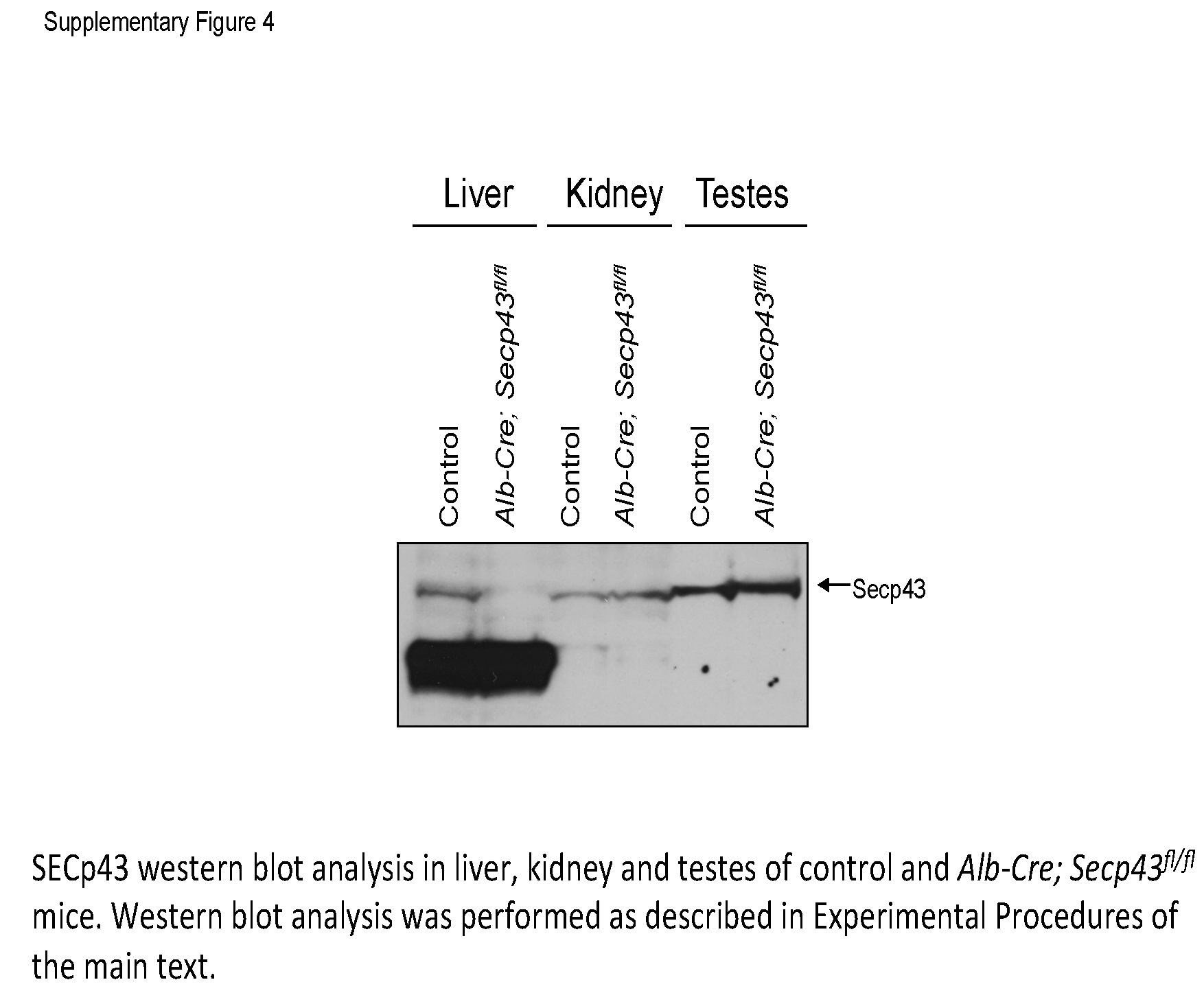

Supplement: S4 Fig — Western blot analysis was performed as described in Experimental Procedures of the main text. (TIFF) [file pone.0127349.s004.tiff]

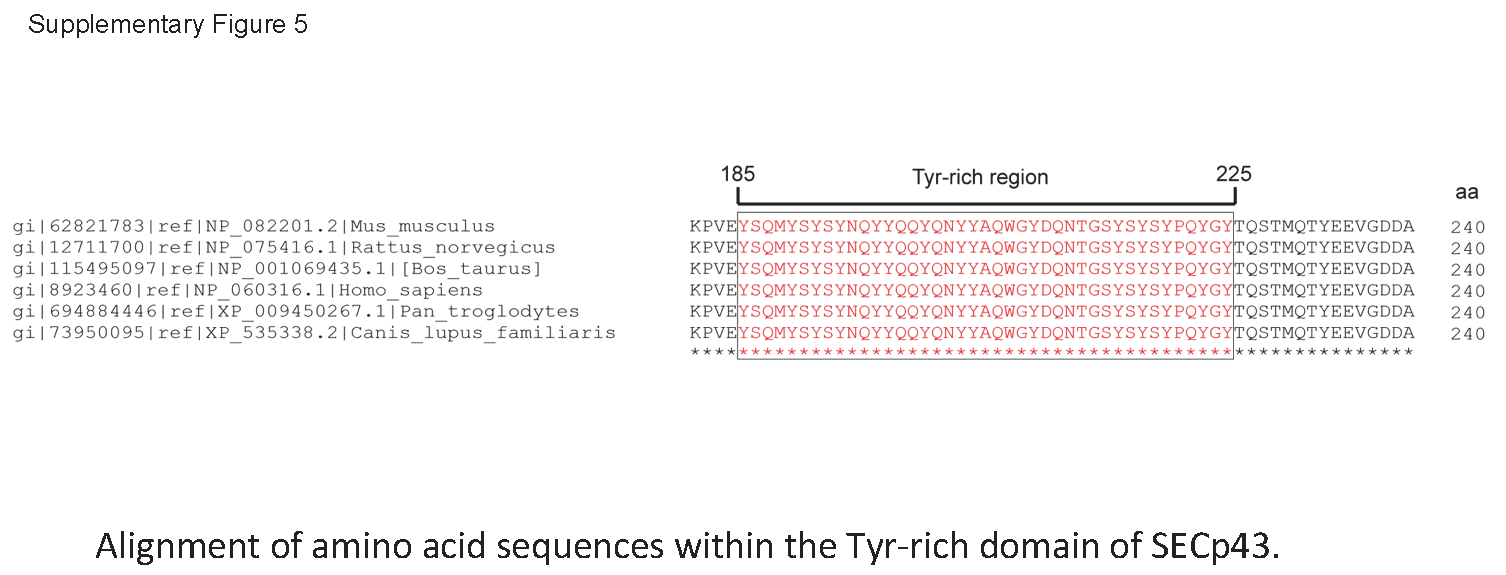

Supplement: S5 Fig — (TIFF) [file pone.0127349.s005.tiff]
